# Herbal Substance, Acteoside, Alleviates Intestinal Mucositis in Mice

**DOI:** 10.1155/2015/327872

**Published:** 2015-01-05

**Authors:** Daniel Reinke, Stamatiki Kritas, Panagiotis Polychronopoulos, Alexios L. Skaltsounis, Nektarios Aligiannis, Cuong D. Tran

**Affiliations:** ^1^Gastroenterology Unit, Women's & Children's Health Network, 72 King William Road, North Adelaide, SA 5006, Australia; ^2^Discipline of Physiology, School of Medical Sciences, University of Adelaide, Adelaide, SA 5005, Australia; ^3^Department of Pharmacognosy & Natural Products Chemistry, National and Kapodistrian University of Athens, 15771 Ilisia, Greece

## Abstract

This study investigated the role of acteoside in the amelioration of mucositis. C57BL/6 mice were gavaged daily with acteoside 600 *μ*g for 5 d prior to induction of mucositis and throughout the experimental period. Mucositis was induced by methotrexate (MTX; 12.5 mg/kg; s.c.). Mice were culled on d 5 and d 11 after MTX. The duodenum, jejunum, and ileum were collected for myeloperoxidase (MPO) activity, metallothionein (MT) levels, and histology. Acteoside reduced histological severity scores by 75, 78, and 88% in the duodenum, jejunum, and ileum, respectively, compared to MTX-controls on d 5. Acteoside reduced crypt depth by 49, 51, and 33% and increased villus height by 19, 38, and 10% in the duodenum, jejunum, and ileum, respectively, compared to MTX-controls on d 5. Acteoside decreased MT by 50% compared to MTX-control mice on d 5. Acteoside decreased MPO by 60% and 30% in the duodenum and jejunum, respectively, compared to MTX-controls on d 5. Acteoside alleviated MTX-induced small intestinal mucositis possibly by preventing inflammation.

## 1. Introduction

Mucositis is characterised by the degradation and increased ulceration of the protective mucosal layer within the gastrointestinal tract (GIT) [1] and occurs in over 40% of all cancer patients that undergo chemotherapy or radiotherapy. Chemotherapeutic agents, such as methotrexate (MTX), attack rapidly dividing cancer cells by inhibiting folate production in RNA and DNA synthesis [1–3]. However, these agents are unable to differentiate between all rapidly dividing cells resulting in destruction of the basal cells and damage to connective tissue in the GIT [3].

Damage to the intestinal lining causes debilitating symptoms [4, 5]. These symptoms are divided into two categories, pain and inflammation. Pain symptoms include loss of eating and eventual anorexia [6]. Inflammation symptoms include diarrhoea, vomiting, and reduced nutrient uptake [7].

The current treatments for mucositis are systemic antibiotics [7–9], anti-inflammatories such as benzydamine and flurbiprofen [7], reducing the immune response and inflammatory markers present in the small intestine [5, 10], and nonspecific pain killers [8] which reduce the amount of damage signalling by nociceptors [11]. Antibiotics decrease levels of bacteria in the small intestine reducing immune mediated inflammation responses [7–9]. These treatments only mask the symptoms but do not treat the condition.

While there are currently no effective treatments for mucositis, many are in development. Numerous novel compounds which may provide preventative protection are being examined, for example, growth factors such as palifermin [7, 8], free radical scavengers such as amifostine [7, 8], or herbal substance such as iberogast [12] and* Rhodiola algida* [13].

The herbal substance, acteoside, is a phenylpropanoid glycoside that is derived from plant species in the* Scrophularia* genus [14, 15]. Plants containing acteoside are often used in traditional medicine to treat a variety of illnesses, such as tumours [16] and inflammation [17]. Hausmann et al. [18] demonstrated that acteoside reduced the intestinal damage caused by experimentally induced inflammatory bowel disease. Acteoside may exert its protective effect through a variety of mechanisms. It is known as an antioxidant [17] that prevents cell apoptosis [19, 20] and sequesters reactive oxygen species (ROS) [21].

It is hypothesised that prophylactic treatment of mice with acteoside will prevent damage caused by MTX to the intestinal mucosa. In the present study, we will be utilising a C57BL/6-mouse model of MTX-induced mucositis to examine the efficacy of acteoside as a potential treatment of mucositis. The mouse model of MTX-induced intestinal mucositis is a well-established model allowing the formation of well-defined, reproducible, clinical, and histological symptoms of mucositis [22].

## 2. Materials and Methods

### 2.1. Animals and Diet

All animal studies were performed in compliance with the Australian Code of Practice for the Care and Use of Animals for Scientific Purposes and were approved by the Animal Ethics Committee of the Women's and Children's Health Network (WCHN). Male C57BL/6 mice (*n* = 64, 7 weeks of age) were housed at room temperature maintained at 25 degrees Celsius with 12-hour dark and light cycles. Mice were given access to standard chow pellets (Specialty Feeds, WA, Australia) and free access to water.

### 2.2. Study Design

**Figure 1:**
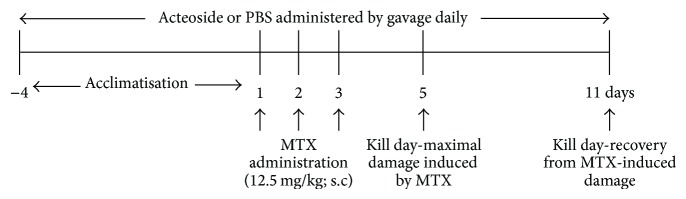
Schematic diagram of the experimental design.

The experiment was undertaken for 16 d, between d −4 and d 11. The first MTX injection was given on d 1. As maximum damage to the small intestinal lining occurs 5 d after the first injection of MTX, mice were culled to assess prevention of damage. Recovery from MTX-induced gut damage occurs from 11 d after the first MTX injection and so this was selected as a cull day to assess the ability of acteoside to enhance intestinal recovery (Figure 1).

### 2.3. Disease Activity Index

The animal's health was recorded using the standard disease activity index (DAI) scoring system and their body weight was recorded daily. DAI is a representative score given for weight loss, general well-being, stool consistency, and blood content in the stool. Categories are rated 1 to 4, with 1 = normal and 4 = severe. Scores from the overall categories are combined for a daily DAI score.

### 2.4. Acteoside, MTX Preparation and Administration

Acteoside (provided by Kapodistrian University of Athens, Greece) powder with purity of >97% was used to prepare a stock solution by suspending 6000 *μ*g dried powder in 1 mL of phosphate buffered saline (PBS). Acteoside (600 *μ*g) was gavage (0.1 mL of 6000 *μ*g/mL) daily throughout the experimental period (Figure 1).

MTX (12.5 mg/kg) was diluted from 50 mg/mL solution (Pfizer Australia Pty Ltd, NSW, Australia) and was injected subcutaneously daily for 3 consecutive days into mice to induce mucositis (Figure 1). Treatments were given to the respective groups by oral gavage. Mice were randomly allocated to 4 groups: PBS + Saline (PS; *n* = 16), Acteoside + Saline (AS; *n* = 16), PBS + MTX (PM; *n* = 16), and Acteoside + MTX (AM; *n* = 16).

### 2.5. Tissue Collection

Mice were culled by asphyxiation with CO_2_ followed by cervical dislocation on d 5 and d 11, after MTX injections (Figure 1). Tissue samples were collected from the duodenum, jejunum, and ileum for histology and biochemical assays (these samples were snap frozen in liquid nitrogen and then stored at −80°C until analysis).

### 2.6. Myeloperoxidase (MPO) Activity

MPO activity in intestinal tissues was determined using a modification of the method by Dahlqvist [23]. Briefly, samples were homogenised (Ultra Turrex homogeniser, Janke and Kunkel, Germany) in 2 mL of saline and centrifuged for 12 min at 15000 rpm (Mikro Benchtop Centrifuge, Hettich GmbH and Co., Tuttlingen, Germany). The supernatant was discarded and the pellet was resuspended in hexadecyltrimethylammonium bromide buffer (HTAB), a detergent (Sigma Aldrich, Sydney, Australia). 50 *μ*L of negative and positive control samples as well as 50 ul of each test sample was added to triplicate wells in a microtiter plate. 200 uL of reaction solution, consisting of 8.4 mg of O-dianisidine dihydrochloride, 24 uL of H_2_O_2_, 5 mL of potassium phosphate buffer (pH 6.0) and 45 mL of distilled H_2_O, was added to all wells in the plate and absorbance was read at 450 nm at 1 min intervals for 15 min with a spectrophotometer (Dynatech MR700, Baxter Diagnostics, Adelaide, South Australia). Results were expressed as units/g of tissue.

### 2.7. Metallothionein (MT) Assay

MT levels in intestinal tissues were determined using a radioactivity ^109^Cd/haem affinity assay as described previously [24]. Briefly, intestinal tissues were homogenized in 10 mM Tris-HCl (pH 8.2) and heated in a boiling water bath for 2 min and then spun at 12,000 g for 2 min. The supernatant was diluted (2 : 1) in 10 mM Tris-HCL and ^109^Cd (Oak Ridge National Laboratory, USA) followed by 4% haemoglobin to mop up excess ^109^Cd. The supernatant was then removed, transferred into a liquid scintillation vial, capped, and analysed using a gamma counter (1282 CompuGamma LRB Wallac, Finland). Results were expressed as nmol of Cd bound/g of wet weight.

### 2.8. Histological Assessment

Intestinal samples were fixed in 10% formalin, processed, embedded in paraffin wax, and then cut and stained with hematoxylin and eosin (H&E) as described previously [25]. Histological assessments were analysed using light microscopes (Olympus, Tokyo, Japan) with an attached video camera (Sony Corporation Tokyo Japan). For villus height and crypt depth, 40 measurements were taken using Image Pro Plus 5.1 software (Media Cybernetics, Maryland, USA). Histological scores were analysed using 11 histological parameters consisting of villus height, crypt depth ratio, enterocyte disruption, goblet cell numbers, mitotic figures, crypt disruption, crypt cell disruption, crypt abscess formation, lymphocytic and polymorphonucleocyte infiltration, capillary and lymphatic dilatation, submucosal thickness, and muscularis thickening. Each score was given a value of 0–3 and combined for an overall score.

### 2.9. Data Analysis

The results were analyzed using SigmaPlot 12.0 (Systat Software, Inc, USA). Two-way ANOVAs with Tukey post hoc tests were used to analyze the results for significance. Statistical significance was considered if *P* < 0.05. Data was expressed as mean ± SEM, unless otherwise stated.

## 3. Results

### 3.1. Disease Activity Index (DAI)

The DAI of the AM group showed no significant difference compared to all other groups throughout the experimental protocol (Table 1). However, the PM group has a significantly (*P* = 0.01) higher DAI compared to the PS group on d 3 and d 5 (Table 1).

### 3.2. MPO Activity

**Figure 2:**
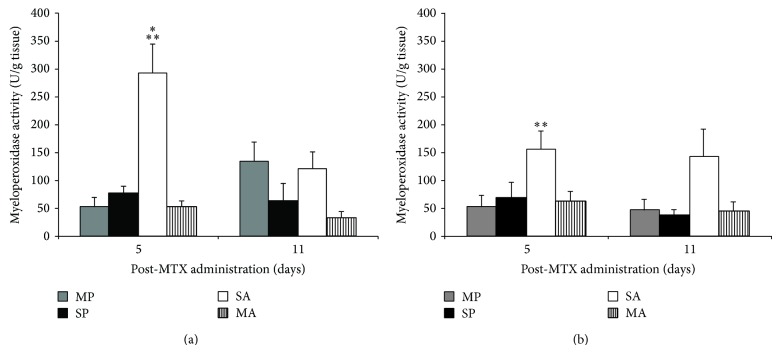
MPO activity (U/g tissue) in the duodenum (a) and jejunum (b) of C57BL/6 mice treated with PS, PM, AS, and MA (*n* = 8/group). ∗∗ indicates significant differences (*P* < 0.01) compared to all other groups on d 5. ∗ denotes significance (*P* < 0.05) compared to PM on d 11.

The AM group showed reduced MPO levels by 59% compared to mice in the PM group (*P* < 0.05) on d 5 (maximal damage) (Figure 2). The mice in the PM group showed increased MPO levels compared to the AS and PS groups within d 5 in jejunum by 60% and the duodenum by 33% (Figure 2).

### 3.3. MT Levels

**Figure 3:**
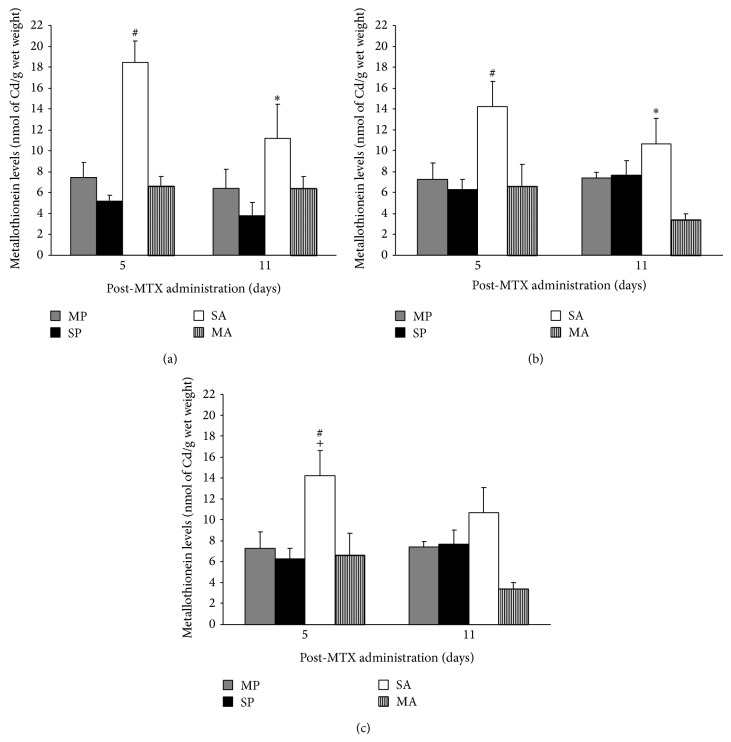
MT levels (nmol Cd bound/g wet weight) in the duodenum (a), jejunum (b), and ileum (c) of C57BL/6 mice treated with PS, PM, AS, and MA (*n* = 8/group). # indicates significant differences (*P* < 0.01) compared to all other groups on d 5. ∗ denotes significance (*P* < 0.05) compared to AS on d 11. + indicates significance (*P* = 0.03) compared to d 5 and d 11 in PM group.

The AM group showed a significant (*P* < 0.05) reduction in MT levels by 64% compared to the mice in the PM group on d 5 (maximal damage) (Figure 3). The PM group showed an increase in MT levels compared to the AS and PS groups within d 5 in all three intestinal segments (Figure 3). The mice in the AM group showed a reduction (*P* < 0.05) in MT levels by 22% compared to the PM group in the duodenum and jejunum only, on d 11 (Figure 3).

### 3.4. Villus Height and Crypt Depth

**Figure 4:**
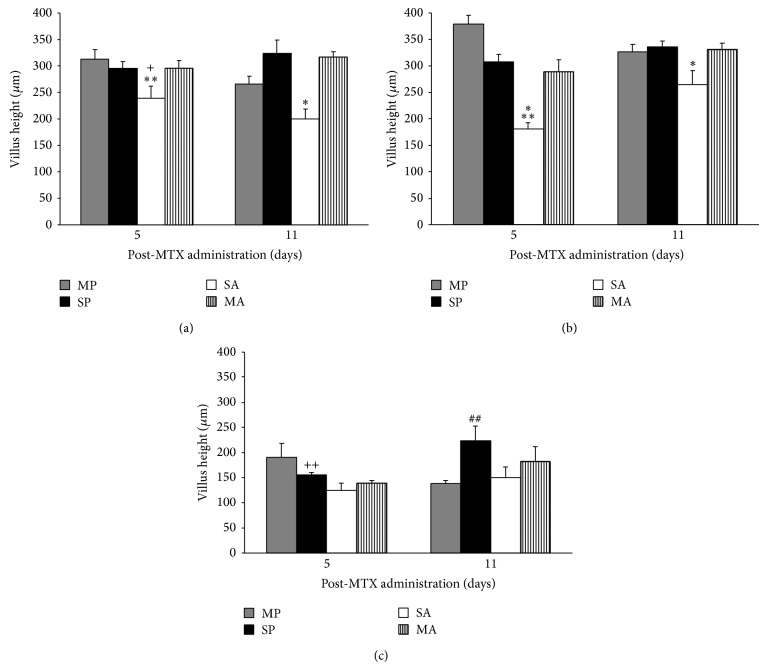
Villus height (μm) in the duodenum (a), jejunum (b), and ileum (c) of C57BL/6 mice treated with PS, PM, AS, and MA. ∗ denotes significant difference (*P* < 0.05) compared to all other groups. ∗∗ denotes significant difference (*P* = 0.027) compared to day 11. + indicates significance (*P* < 0.01) compared to PS on d 5. ## indicates significant difference compared to PS and PM groups on d 11. ++ indicates significant difference (*P* = 0.015) compared to d 11.

**Figure 5:**
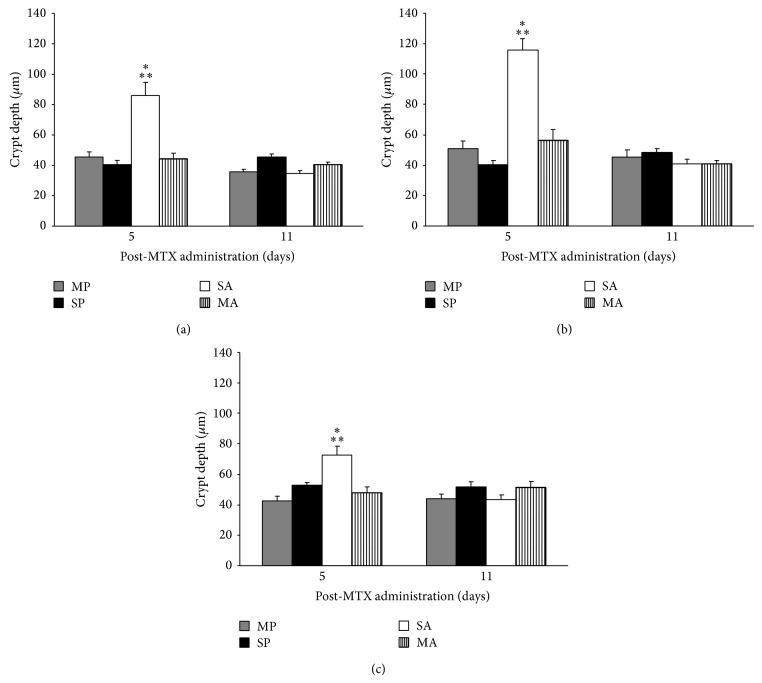
Crypt depth (*μ*m) in the duodenum (a), jejunum (b), and ileum (c) of C57BL/6 mice treated with PS, PM, AS, and MA. ∗ denotes significant difference (*P* < 0.01) compared to all other groups within d 5. ∗∗ denotes significant difference (*P* < 0.01) compared to d 11.

The PM group showed a significant increase in crypt depth and decrease in villus height on d 5 compared to all other groups in the duodenum and jejunum (Figures 4 and 5). Crypt depth and villus height were significantly (*P* < 0.05) increased by 49, 51, and 33% and decreased by 19, 38, and 10%, respectively, in the PM group on d 5 compared to d 11 (Figures 4 and 5).

### 3.5. Histological Severity Scores

**Figure 6:**
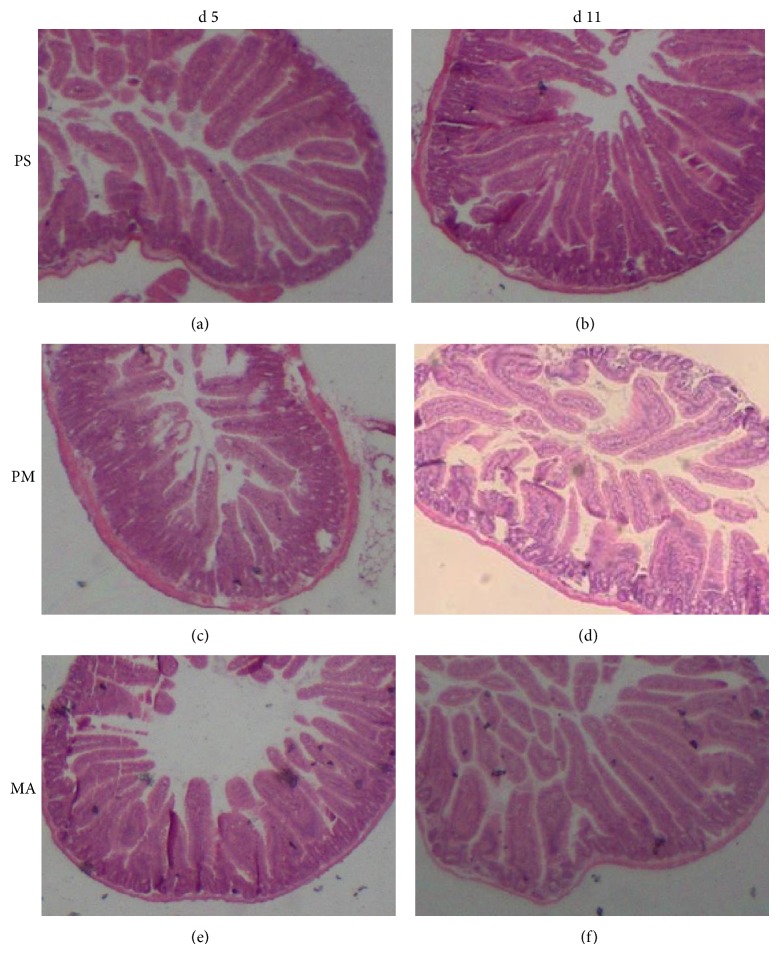
Histological sections of the jejunum stained with H&E taken at 20x magnification. Images (a) and (b) represent the PS group (control) for d 5 and d 11, respectively, with normal villi and crypts. Images (c) and (d) represent the PM group (MTX-treated) for d 5 and d 11, respectively; the administration of MTX was characterized by atrophy of the villi, increased crypt depth, and enterocyte disruption, predominantly on d 5. Images (e) and (f) represent the MA group (MTX-treated + acteoside) for d 5 and d 11, respectively; the administration of acteoside administration prevented villus and crypt damage on d 5 and d 11.

PM treated mice had a significantly increased (88%) histological severity score on d 5 compared to all other groups (Table 2; Figure 6). Histological severity scores were significantly (*P* < 0.05) decreased in the PM treated group on d 11 compared to d 5 (Table 2; Figure 6). Mice treated with PM showed a decreased infiltration of inflammatory cells and increased mitotic cells present as well as a reduction in damage to cell structure compared to AM and PB treated mice on d 5 (Figure 6).

## 4. Discussion

The current study investigated the protective effect of acteoside, a herbal substance, on a mouse model of intestinal mucositis. Acteoside is known for its antioxidative properties as well as its ability to reduce inflammation in multiple tissues [19]. The administration of 3 consecutive doses of MTX (daily) induced a mild mucositis which was observed on d 5 as indicated by increased MPO activity and histological severity scores and this is consistent with previous studies [3–5, 25].

DAI is indicative of the severity of intestinal damage and mice treated with acteoside showed a trend towards a reduction in the severity of clinical symptoms caused by MTX. The trend represented in the DAI scores was consistent with the results observed from the severity scores suggesting that the DAI may be a good indicator of mucosal damage. Furthermore, we have shown that acteoside may be protective against MTX-induced mucositis as supported by our data of reduced severity scores, crypt depth, MPO activity, and MT levels.

Based on our histological severity score parameters, it was observed that degradation of the cellular structure and shape of the cells present in the mucosal lining was reduced. No noticeable changes were observed in mucosal thickening or vascularisation. The dividing cells in the base of the crypts were protected and the number of inflammatory cells present in the mucosa were reduced, consistent with previous studies [21, 25]. The severity scores suggested that acteoside may have a protective action on the small intestine mucosal lining. Furthermore, crypt depth on d 5 was reduced while villus height was increased in mice given acteoside. This indicated the possibility of a protective action on the cells in the villus and crypts. This may be due to a decrease in the production of TNF-*α* which, in turn, reduces the inflammation by preventing apoptosis of the stem cells in the crypts [26]. We have also shown that the administration of acteoside reduced MPO activity in mice treated with MTX indicating that acteoside may reduce MTX-induced inflammation.

The administration of acteoside markedly reduced MT levels in mice treated with MTX compared to mice treated with MTX alone. MT, an intracellular heavy metal binding, cystine rich ligand, has the ability to bind ROS [27]. The findings suggest that the endogenous MT may have been used to bind and break down ROS associated with MTX-induced mucositis which has been postulated to be the primary reason for reducing inflammation [28]. However, it is unclear how this is achieved; interestingly, we found that acteoside alone did not induce MT levels and there are no supporting studies indicating that acteoside upregulates MT. Further research into this pathway is warranted.

However, it has been shown that acteoside reduces levels of TNF-*α* [26], cell mediated apoptosis, and the production of ROS is regulated by TNF-*α* [29–34]. The reduction of TNF-*α* by acteoside means that local inflammation and cell mediated apoptosis are reduced by the presence of acteoside. This forms a possible mechanism for the function of acteoside in reducing intestinal damage by MTX. Acteoside is known to have antiapoptotic properties [18]. Its ability to inhibit the production of ROS inside cells enables it to prevent the apoptosis of cells [14]. This process suggests that acteoside is able to reduce TNF-*α* levels within the cell, which is a marker essential for cell mediated death [14, 26]. This implies that the preventative treatment of mice with acteoside may inhibit the initial lysing of basal stem cells in the small intestine in the short term preventing the initial change in tissue response.

The findings from the present study did not show an improvement in recovery rate with respect to MTX-induced damage with the administration of acteoside. This may be expected as acteoside has been shown to partially prevent MTX-induced damage on d 5, and an increased recovery rates may not be observable. It is possible that the protection to the mucosal lining by acteoside was sufficient in the early phase of damage so that recovery rate from damage played a minor role. However, it is possible that acteoside functions in such a way as to prevent increased inflammation but has no direct effect on enhancing the rate of recovery of cells that have already been damaged.

In conclusion, mice treated with MTX to induce mucositis showed reduced levels of damage and inflammation to the mucosal lining when given acteoside prophylactically. Acteoside may be protective against MTX-induced small intestinal damage, by a couple of mechanisms, firstly increasing mitotic cells, thereby increasing cell proliferation, and secondly reducing inflammatory cell numbers as indicated by decreased MPO activity. Acteoside appears to be a promising alternative therapeutic approach for treating chemotherapy-induced mucositis.

## Figures and Tables

**Table 1:** Disease activity index of C57BL/6 mice (*n* = 8/group) treated with PS, PM, AS, and MA.

Treatment	d −4	d 1	d 2	d 3	d 5	d 11
PS	1 (1, 1)	2 (1, 2)	1 (1, 2)	1 (1, 2)	1 (1, 2)	1 (1, 1)
AS	1 (1, 1)	1 (1, 2)	2 (1, 4)	1.5 (1, 2)	1 (1, 1)	1 (1, 1)
PM	1 (1, 1)	2 (1, 2)	2 (1, 4)	3 (2, 4)^*^	2 (1, 3)^*^	1 (1, 1)
MA	1 (1, 1)	1 (1, 2)	2 (1, 4)	2 (1, 3)	1.5 (1, 2)	1 (1, 1)

Data was expressed as median and range in parenthesis. ^*^Statistical significance *P* = 0.01 compared to PS within group. PS = PBS + saline, AS = acteoside + saline, PM = PBS + MTX, and MA = MTX + acteoside.

**Table 2:** Histological severity scores for C57BL/6 mice (*n* = 8/group) treated with PS, PM, AS, and MA.

Treatment	Duodenum		Jejunum		Ileum	
	d 5	d 11	d 5	d 11	d 5	d 11
PS	2.5 (2, 5)	3 (1, 6)	2.5 (1, 4)	5 (2, 9)	3 (1, 5)	1 (1, 5)
AS	2.5 (1, 3)	2 (1, 6)	3 (2, 7)	3 (2, 4)	1.5 (1, 4)	2 (0, 2)
PM	13 (2, 16)^*^	2 (1, 4)^#^	13 (10, 18)^*^	6 (2, 10)^#^	13 (7, 15)^*^	1 (0, 3)^#^
MA	3 (2, 5)	2.5 (1, 6)	2.5 (2, 4)	3 (1, 8)	1 (1, 3)	1 (1, 4)

Data was expressed as median and range in parenthesis. ^*^Statistical significance compared to all other groups (*P* < 0.01) on d 5. ^#^Statistical significance (*P* < 0.01) compared to d 5. PS = PBS + saline, AS = acteoside + saline, PM = PBS + MTX, and MA = MTX + acteoside.
